# Observing the distribution of mosquito bites on humans to inform personal protection measures against malaria and dengue vectors

**DOI:** 10.1371/journal.pone.0271833

**Published:** 2022-07-25

**Authors:** Winifrida P. Mponzi, Johnson K. Swai, Emmanuel W. Kaindoa, Khamis Kifungo, Alvaro E. Eiras, Elis P. A. Batista, Nancy S. Matowo, Peter O. Sangoro, Marceline F. Finda, Arnold S. Mmbando, Tegemeo Gavana, Halfan S. Ngowo, Fredros O. Okumu

**Affiliations:** 1 Environmental Health and Ecological Science Department, Ifakara Health Institute, Ifakara, Tanzania; 2 School of Public Health, Faculty of Health Sciences, University of the Witwatersrand, Johannesburg, South Africa; 3 The Nelson Mandela, African Institution of Science and Technology, School of Life Sciences and Bio Engineering, Arusha, United Republic of Tanzania; 4 Department of Parasitology, Laboratory of Technological Innovation of Vectors Control, Biological Sciences Institute, Federal University of Minas Gerais Belo Horizontal Brazil, Belo Horizonte, Brazil; 5 Department of Disease Control, London School of Hygiene and Tropical Medicine, London, United Kingdom; 6 International Centre of Insect Physiology and Ecology, Nairobi, Kenya; 7 Department of Biosciences, Durham University, Durham, United Kingdom; 8 Institute of Biodiversity, Animal Health and Comparative Medicine, University of Glasgow, United Kingdom; Al-Azhar University, EGYPT

## Abstract

**Background:**

Understanding mosquito biting behaviours is important for designing and evaluating protection methods against nuisance biting and mosquito-borne diseases (e.g. dengue, malaria and zika). We investigated the preferred biting sites by *Aedes aegypti* and *Anopheles arabiensis* on adult volunteers in standing or sleeping positions; and estimated the theoretical protection limits affordable from protective clothing or repellent-treated footwear.

**Methods:**

Adult volunteers dressed in shorts and t-shirts were exposed to infection-free laboratory-reared mosquitoes inside screened chambers from 6am to noon (for day-biting *Ae*. *aegypti*) or 6pm to midnight (night-biting *An*. *arabiensis*). Attempted bites on different body parts were recorded. Comparative observations were made on same volunteers while wearing sandals treated with transfluthrin, a vapour-phase pyrethroid that kills and repels mosquitoes.

**Results:**

*An*. *arabiensis* bites were mainly on the lower limbs of standing volunteers (95.9% of bites below the knees) but evenly-distributed over all exposed body surfaces when the volunteers were on sleeping positions (only 28.8% bites below knees). *Ae*. *aegypti* bites were slightly concentrated on lower limbs of standing volunteers (47.7% below knees), but evenly-distributed on sleeping volunteers (23.3% below knees). Wearing protective clothing that leave only hands and head uncovered (e.g. socks + trousers + long-sleeved shirts) could theoretically prevent 78–83% of bites during sleeping, and at least 90% of bites during non-sleeping hours. If the feet are also exposed, protection declines to as low as 36.3% against *Anopheles*. The experiments showed that transfluthrin-treated sandals reduced *An*. *arabiensis* by 54–86% and *Ae*. *aegypti* by 32–39%, but did not change overall distributions of bites.

**Conclusion:**

Biting by *An*. *arabiensis* and *Ae*. *aegypti* occur mainly on the lower limbs, though this proclivity is less pronounced in the *Aedes* species. However, when hosts are on sleeping positions, biting by both species is more evenly-distributed over the exposed body surfaces. High personal protection might be achieved by simply wearing long-sleeved clothing, though protection against *Anopheles* particularly requires covering of feet and lower legs. The transfluthrin-treated footwear can reduce biting risk, especially by *An*. *arabiensis*. These findings could inform the design and use of personal protection tools (both insecticidal and non-insecticidal) against mosquitoes and mosquito-borne diseases.

## Background

Vector-borne diseases are widespread across the globe and are a major cause of public health and economic failures affecting millions. The most prevalent of these diseases are malaria and dengue fever, which are transmitted by *Anopheles* and *Aedes* mosquitoes, respectively [1] While vector control has contributed significantly to malaria control in Africa [2], both dengue fever and other *Aedes-*borne diseases remain highly neglected in the continent. Despite recent successes with the use of *Wolbachia* endosymbionts [3], the control of *Aedes*-borne viruses still relies mostly on personal protection measures [4, 5].

Successful transmission of mosquito-borne pathogens is mediated by the blood-feeding habits of female mosquitoes, which may express preferences for specific blood hosts [6]. To acquire a blood meal, the host-seeking females must successfully locate and bite their hosts. They identify human hosts by detecting specific cues in the environment before biting at selected sites [7]. The mosquitoes rely on a variety of environmental and host-derived stimuli such as visual cues, moisture, heat, carbon dioxide and odours from skin emanations [8, 9]. Since female mosquitoes depend on blood meals for eggs development [10], these man-vector contacts are a vital component of the disease transmission process. Efficient vectors of human pathogens therefore tend to live near humans and can develop high degrees of anthropophily and anthropophagy.

Once mosquitoes have reached humans, their actual landing sites and the resulting distribution of biting are evidently non-random, as some body parts receive more bites than others [11]. It has been shown that malaria vectors, such as *Anopheles arabiensis*, *An*. *funestus*, *An*. *gambiae* often bite mostly on the feet and ankles of people sitting upright but this preference diminishes when people lie down [9, 11, 12]. On the other hand, *Aedes aegypti*, *Ae*. *simpsoni* and *Ae*. *atroparvus* prefer biting around the head and shoulders [8], while *Ae*. *albopictus* prefers biting around the feet [13]. Whilst scale up of the core vector control tools, long-lasting insecticidal nets (LLINs) and indoor residual sprays (IRS) have reduced the burden of mosquito-borne diseases such as malaria [2, 14] further progress is hampered by several factors, among them, the rise of physiological [15] and behavioural resistance [16, 17]. This calls for additional protection, including those suitable for use outdoors and when people are outside bed nets [18]. Passive spatial repellents are being developed to address these gaps and have the advantage of simultaneously protecting multiple people by deterring, inhibiting feeding, and at times killing mosquito vectors [19–21].

In a recent study by Braack *et al* [11], who demonstrated the differential bite distribution of mosquito species over volunteer bodies, the authors recommended that certain forms of protection such as protective clothing and insecticide-treated footwear may reduce biting risk. Since then, prototype repellent sandals have been demonstrated to reduce overall biting risk under experimental conditions [22] though no studies have been done to illustrate whether the sandals influence mosquito behaviours. Such sandals, if used alongside ITNs, have the potential of conferring round-the-clock protection since footwear are already commonplace and are used most times.

This current study therefore investigated the preferred biting sites by both the dengue vector, *Ae*. *aegypti* and the malaria vector, *An*. *arabiensis* on adult male volunteers in standing or sleeping positions, and further estimated protection limits affordable by either protective clothing or the repellent-treated footwear.

## Methods

### Semi-field system

**Fig 1:**
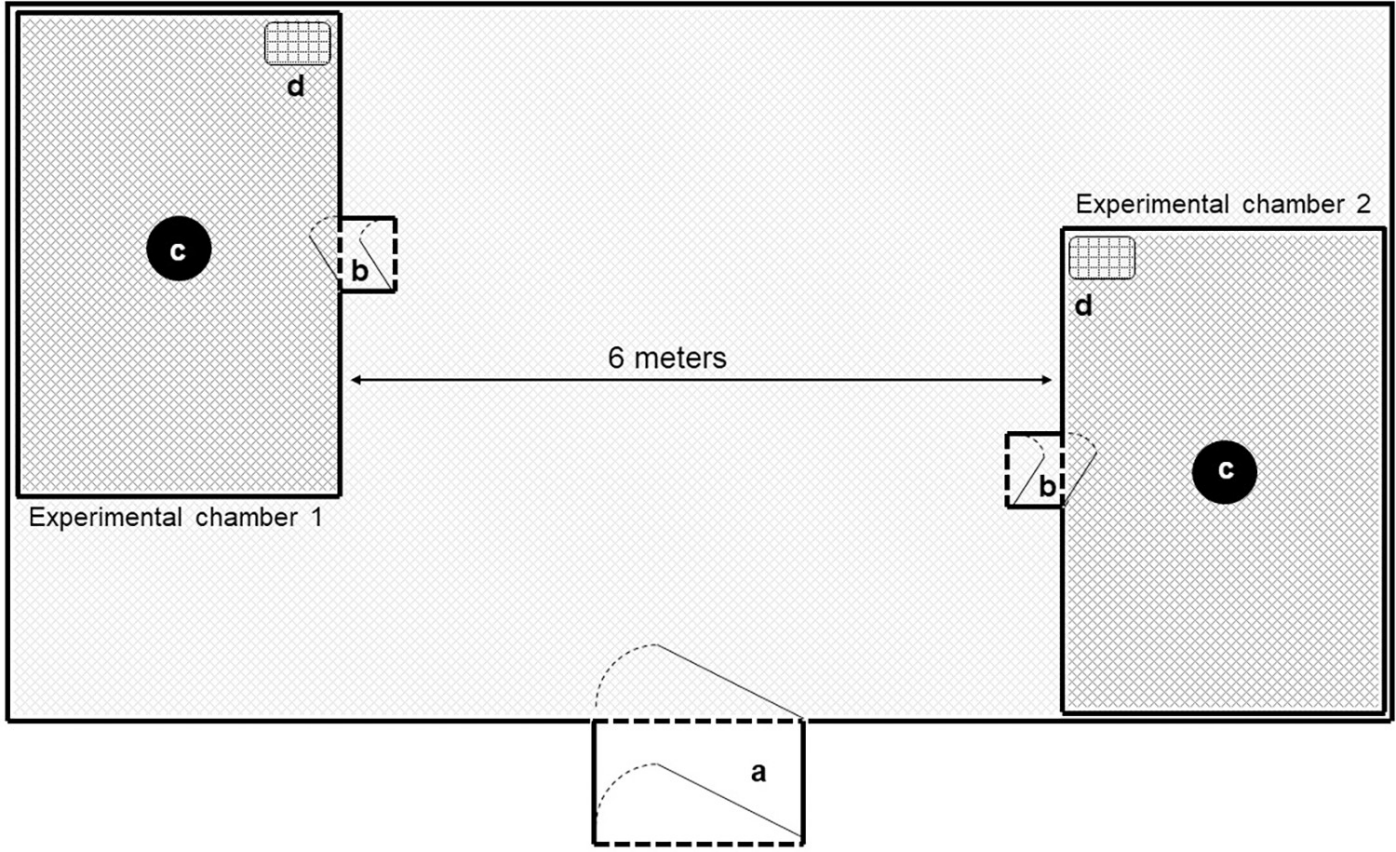
Illustration of the experimental chambers inside the semi-field facility, showing: a) Entry into the main chamber; b) Entries into the experimental cages; c) Volunteer stations and d) Mosquito release points.

The study was conducted at the Ifakara Health Institute’s semi-field facility located in Ifakara, Tanzania. The facility has three chambers each measuring 9.6m wide × 21m long) [23], and one of which was used for this study. Two large experimental cages (6m wide × 6m long × 2.8m high), made of fiberglass netting and PVC flooring were erected 6m apart inside the semi-field chamber (Fig 1). It is inside these fibreglass netting cages that the actual experiments were conducted.

### Mosquitoes

Laboratory-reared nulliparous 4–9 days old *An*. *arabiensis*, and *Ae*. *aegypti* mosquitoes, starved for six hours prior to experimentation, were used. These infection-free mosquito colonies were maintained using standard procedures as previously described [23, 24].

### Study volunteers

Adult male volunteers (25–36 years old) were involved in the study. The volunteers were recruited upon providing a written informed consent once the purpose, benefits and potential risks of the study had been explained to them. They were instructed not to use any fragranced soap or perfume, tobacco or alcohol throughout the experiment period.

### Transfluthrin-treated sandals

**Fig 2:**
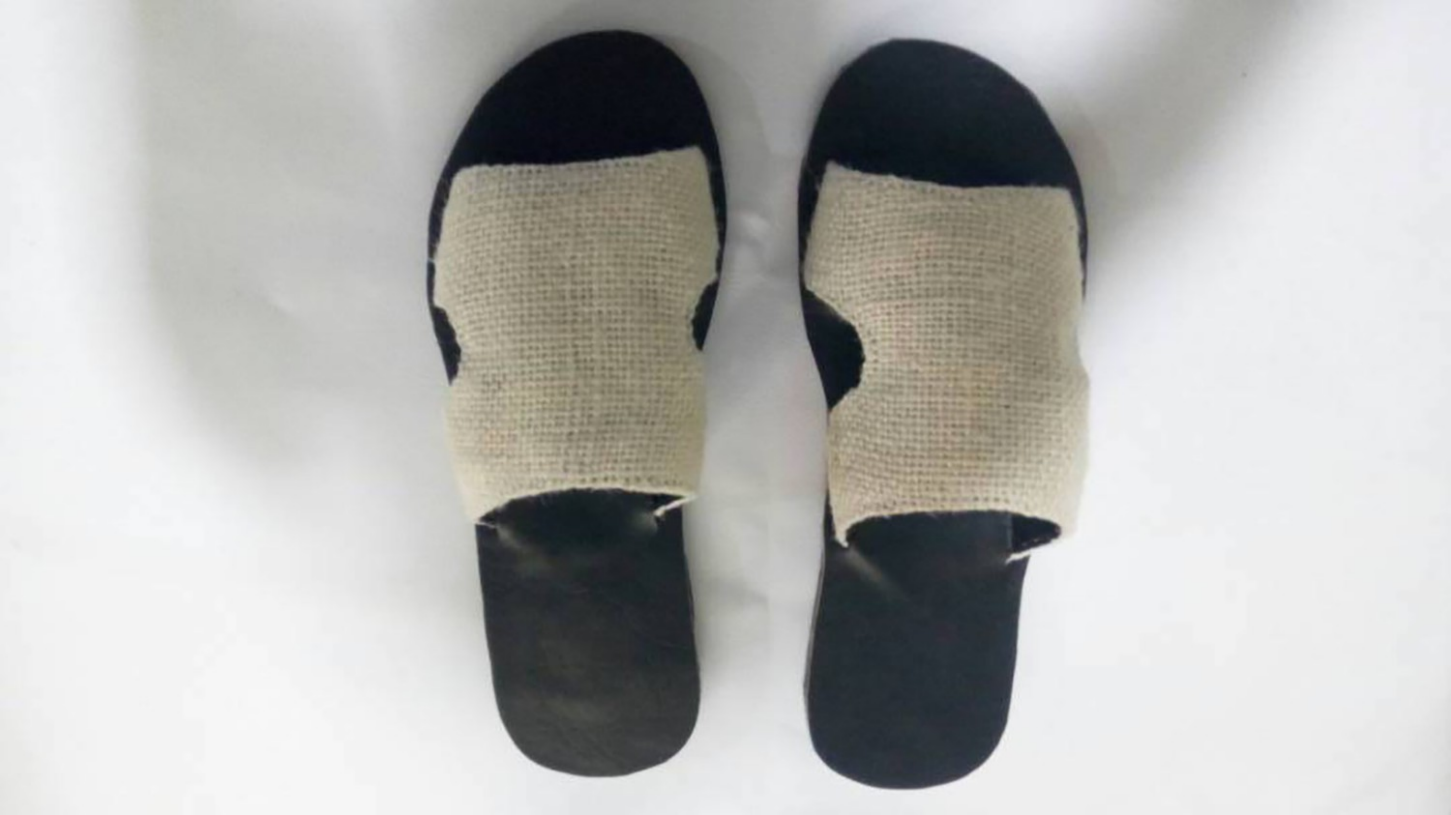
A pair of transfluthrin-treated sandals.

We used the modified design of repellent-treated sandals from previously described by Sangoro *et al* [22]. The sandals had an active surface area of 395cm^2^ each and were made of hessian (Fig 2). They were treated using a 10% transfluthrin solution to achieve 0.04 g/cm^2^. The treated sandals were dried and wrapped in aluminium foil, and were stored after every experiment. The sandals were stored under the shade to minimize the wear out of the insecticide.

### Study design

Four volunteers working in two pairs were involved in the experiment. Two of the volunteers were the actual test subjects, while the other two collected mosquitoes landing on the test subjects. These volunteers were recruited upon consent and trained on the specific procedures for this study. The volunteer pairs occupied separate large cages (Fig 1) and worked together throughout the experiment. In each pair of volunteers, the test subject wore only short trousers and a short-sleeved t-shirt, while the second volunteer, i.e. the mosquito collector, wore closed shoes, long-sleeved shirt, long-trousers and gloves to prevent mosquito bites. This fully-clothed volunteer monitored and collected the mosquitoes landing on his colleague, the test subject. Using this approach, we observed the distribution of landing sites of the released mosquitoes on the bodies of the test volunteers when they were either lying down horizontally on a flat bed or standing upright. The experiments were completed with the test volunteers either wearing or not-wearing a transfluthrin treated sandal.

Observations were done for six hours starting either early morning (for day-biting *Ae*. *aegypti;* 06:00am to 12:00 noon) or early evening (for night-biting *An*. *arabiensis*; 06:00pm to 12:00 mid-night). During each experimental replicate, 100 sugar-starved female mosquitoes (4–9 day old nulliparous) were released in each of the experimental cages (Fig 1), 50 mosquitoes at the beginning and another 50 after three hours. The first releases were done just before the volunteers entered the chambers and were left for 10 minutes to acclimatize with the environment. The volunteers collected mosquitoes for 45 minutes and rested for 15 minutes of each hour of the experiment. The collector tallied all observed mosquito landings by body part. The landing mosquitoes were captured using mouth aspirators and their locations considered a proxy for actual biting sites.

The observations were replicated for at least 20 days (*Ae*. *aegypti*) and 20 nights (*An*. *arabiensis*) for each of the following set-ups: a) tests involving volunteers in sleeping position and wearing untreated sandals, b) tests involving the volunteers in standing position and wearing untreated sandals, c) tests involving the volunteers in sleeping position and wearing treated sandals, and d) tests involving the volunteers in standing position and wearing treated sandals. The tests without treated sandals were completed before the tests with treated sandals.

### Data analysis

Data were analysed using Stata^®^ 15.1 (College Station, TX, USA). Frequencies, percentages, means and 95% confidence intervals were estimated to describe the distribution of mosquito landings on the volunteers for the two species, when the volunteer was either on a sleeping position or upright, as well as when the volunteer, in either of the two positions, was wearing untreated sandals or transfluthrin-treated sandals. A paired t-test was performed to compare bites occurring on different human body parts when the volunteer was lying down and standing upright with untreated sandals and wearing transfluthrin-treated sandals. In this analysis, we counted only the mosquito that were landing on the volunteers who were the test subject of the experiment. The collectors remained fully clothed during the experiments and did not experience any bites, thus they were not included in the analysis.

### Ethical consideration

Ethical approval was provided by Institutional Review Board (IRB) of Ifakara Health Institute approval number IHI/IRB/NO: 10–2017; and the Medical Research Coordinating Committee of the National Institute for Medical Research, in Tanzania with approval number NIMR/HQ/R.8a/VOL1X/2555. The permission to publish this study was granted by director general of National Institute of Medical Research in Tanzania (Ref: NIMR/HQ/P.12 VOL XXXIV/18)

## Results

### General distribution of biting sites by *Ae*. *aegypti*

Densities of the observed *Ae*. *aegypti* landings (as a proxy for biting) are summarised in Table 1. A total of 4,065 mosquitoes were recaptured in tests where the volunteers were in a sleeping position, and 5,848 mosquitoes recaptured in tests with volunteers in standing position. When in a sleeping position, most of the host-seeking *Ae*. *aegypti* mosquitoes landed on the forearms (26.8%) and the rest were distributed generally evenly over the rest of the exposed body surfaces. Cumulative estimates from foot to head show that only 23% of the landings were below the knee (Table 1). On the other hand, when the volunteers were on a standing position, most landings were on the legs followed by forearms, with 47.7% of landings below the knees.

**Table 1:** Distribution of *Aedes aegypti* landing sites on bodies of volunteers in sleeping and standing positions.

		Volunteers in sleeping position				Volunteers in standing position				
Body part	N	Recaptured	Mean [95% CI]	Distribution (Percentage)	Cumulative Distribution (%)	Recaptured	Mean [95% CI]	Distribution (Percentage)	Cumulative Distribution (%)	Paired t-test p-value
Head	80	522	6.5 [5.5–7.6]	12.8	100.0	291	3.6 [3.0–4.3]	5.0	100.0	<0.001
Hand	80	361	4.5 [3.8–5.2]	8.9	87.2	290	3.6 [2.9–4.3]	5.0	95.0	0.0769
Fore arm	80	1,089	13.6 [12.0–15.2]	26.8	78.3	995	12.4 [11.3–13.6]	17.0	90.1	0.2143
Upper arm	80	467	5.8 [4.7–7.0]	11.5	51.5	332	4.2 [3.4–4.9]	5.7	73.1	0.0115
Torso	80	155	1.9 [1.4–2.5]	3.8	40.0	329	4.1 [3.3–4.9]	5.6	67.4	<0.001
Upper leg	80	534	6.7 [5.4–7.9]	13.1	36.2	821	10.3 [9.0–11.5]	14.0	61.7	<0.001
Lower leg	80	613	7.7 [6.3–9.1]	15.1	23.1	1,713	21.4 [18.8–24.0]	29.3	47.7	<0.001
Foot	80	324	4.1 [2.8–5.3]	8.0	8.0	1,077	13.5 [10.9–16.1]	18.4	18.4	<0.001
		**4065**				**5848**				**-43.9%**

Generally, there were 44% fewer mosquitoes observed on volunteers while on standing compared to sleeping positions. The paired t-tests revealed significantly fewer landings on standing volunteers for all exposed body parts except hand (p = 0.078), forearms (p = 0.2143) and upper arm (p = 0.0115).

### General distribution of biting sites by *An*. *arabiensis*

Results for biting distribution by *An*. *arabiensis* are summarized in Table 2. A total of 2,754 mosquitoes were recaptured in tests where the volunteers were in a standing position, and 3,057 when the volunteers were in sleeping position. In the sleeping position, most of the host-seeking *An*. *arabiensis* landed on the forearms (26.7%) and the rest were distributed evenly over other exposed body surfaces. Only 28.8% of the landings were below the knee (Table 2). On the other hand, when the volunteers were standing, nearly all the landings occurred on the legs (95.9% on the foot and lower leg). There was a 10% increase in mosquitoes landing on standing volunteers compared to sleeping volunteers. The paired t-tests revealed significantly more landings on standing volunteers for all exposed body parts (p ≤ 0.01).

**Table 2:** Distribution of *An*. *arabiensis* landing sites on bodies of volunteers in sleeping and standing positions.

		Volunteers in sleeping position				Volunteers in standing position				
Body part		Recaptured	Mean [95% CI]	Distribution (Percentage)	Cumulative Distribution (%)	Recaptured	Mean [95% CI]	Distribution (Percentage)	Cumulative Distribution (%)	Paired t-test
										p-value
Head	80	169	2.1 [1.6–2.6]	5.5	100.0	2	0.0 [0.0–0.1]	0.1	100.0	<0.001
Hand	80	359	4.5 [3.5–5.4]	11.7	94.5	2	0.0 [0.0–0.1]	0.1	99.9	<0.001
Fore arm	80	817	10.2 [8.9–11.5]	26.7	82.7	21	0.3 [0.0–0.5]	0.8	99.9	<0.001
Upper arm	80	302	3.8 [2.9–4.6]	9.9	56.0	0	0	0.0	99.1	<0.001
Torso	80	111	1.4 [1.0–1.8]	3.6	46.1	1	0	0.0	99.1	<0.001
Upper leg	80	418	5.2 [4.1–6.4]	13.7	42.5	88	1.1 [0.5–1.7]	3.2	99.1	<0.001
Lower leg	80	594	7.4 [6.0–8.9]	19.4	28.8	891	11.1 [8.3–14.0]	32.4	95.9	0.0087
Foot	80	287	3.6 [2.6–4.6]	9.4	9.4	1,749	21.9 [16.9–26.8]	63.5	63.5	<0.001
		**3057**				**2754**				**9.9%**

### Theoretical estimates of protection by protective clothing

We have estimated these theoretical protection measures from Tables 1 and 2, assuming that clothing, even if non-insecticidal will directly prevent bites on the covered surfaces. *An*. *arabiensis* bites were mainly on the lower limbs of standing volunteers. In this case, up to 95.9% were below the knees suggesting that protective clothing on these regions would greatly reduce biting. However, biting by the same species were evenly-distributed over all exposed body surfaces when the volunteers were on sleeping positions, as only 28.8% of the bites were below knees. For *Ae*. *aegypti*, the bites were marginally concentrated below the knees of standing volunteer, but evenly-distributed on sleeping volunteers.

Based on these estimates and the assumptions of physical barrier protection, wearing protective clothing that leave only hands and head uncovered, for example socks + trousers + long-sleeved shirts, can prevent 78–83% of all bites during sleeping, and 90–99.9% of all bites during non-sleeping hours. However, if the feet are also exposed (e.g. when people are wearing sandals and no socks, then the protection declines to as low as 36.3% against *Anopheles* during non-sleeping hours and to 70–73% in all other tested situations with either *Anopheles* in sleeping position or *Aedes* in both sleeping and non-sleeping positions.

### Effect of transfluthrin-treated sandals on biting distribution and densities

For *Ae*. *aegypti* approaching a sleeping volunteer, transfluthrin-treated sandals reduced potential bites over the whole body by 38.8%, without markedly changing the actual distribution of the landing sites relative to sleeping volunteers with untreated sandals. Statistically-significant reductions were observed on the feet (by 98%; p<0.001), lower leg (74%; p<0.001), upper leg (58%; p<0.001) and hands (36%; p = 0.0065), but not head or upper arms (Table 3). We also observed the change in biting pattern by *Ae aegypti* when the volunteer wore sandals, as most of the bites shifted from the lower limb to the upper legs and torso. Similarly, the transfluthrin-treated sandals reduced overall *Ae*. *aegypti* landings on standing volunteers by 32.2%, though statistically significant reductions occurred only on the lower legs (38%; p<0.001) and feet (78%; p<0.001) (Table 3).

**Table 3:** Mean number of *Aedes aegypti* caught at different body parts of volunteers with and without the transfluthrin-treated sandals.

**Body part**	**N**	**Volunteers on a sleeping position**					**Paired t-test p-value**		** **
		**Without Sandals**		**With Sandals**				**% Reduction**	
		**Recaptured**	**Mean [95% CI]**	**Recaptured**	**Mean [95% CI]**				
**Head**	40	221	5.5 [4.1–6.9]	301	7.5 [5.9–9.1]	0.0602		-36	
**Hand** ^**++**^	40	220	5.5 [4.3–6.6]	141	3.5 [2.8–4.2]	0.0065		36	
**Fore arm**	40	600	15 [12.9–17.1]	489	12.2 [9.9–14.6]	0.0775		19	
**Upper arm**	40	226	5.7 [3.8–7.5]	241	6.0 [4.6–7.4]	0.7523		-7	
**Torso**	40	76	1.9 [1.0–2.8]	79	2.0 [1.3–2.6]	0.9039		-4	
**Upper leg** *	40	375	9.4 [7.5–11.2]	159	4.0 [2.7–5.2]	<0.001		58	
**Lower leg** *	40	485	12.1 [10.3–13.9]	128	3.2 [2.3–4.1]	<0.001		74	
**Foot** *	40	319	8.0 [6.3–9.7]	5	0.1 [0.0–0.2]	<0.001		98	
		**Volunteers on standing position**							
	N	**Without Sandals**		**With Sandals**		**Paired t-test p-value**		**% Reduction**	
		**Recaptured**	**Mean [95% CI]**	**Recaptured**	**Mean [95% CI]**				
**Head**	40	147	3. 7 [2.7–4.6]	144	3.6 [2.8–4.4]	0.8758		2	
**Hand**	40	167	4.2 [3.0–5.3]	123	3.1 [2.3–3.8]	0.0645		26	
**Fore arm**	40	521	13.0 [11.4–14.7]	474	11.9 [10.2–13.5]	0.2462		9	
**Upper arm**	40	175	4.3 [3.2–5.5]	157	3.9 [3.0–4.8]	0.5026		10	
**Torso**	40	160	4.0 [3.0–5.0]	169	4.2 [2.9–5.5]	0.7555		-6	
**Upper leg**	40	375	9.4 [7.8–11.0]	446	11.2 [9.1–13.2]	0.156		-19	
**Lower leg** *	40	1,058	26.5 [23.3–29.6]	655	16.4 [12.8–19.9]	<0.001		38	
**Foot** *	40	882	22.1 [18.8–25.3]	195	4.9 [3.4–6.3]	<0.001		78	

^**#**^ p≤ 0.05

^**++**^ p≤0.01

* p≤0.001. Reporting the two-sided p-value of paired T-test.

**N–**total number of replicates, we had two volunteers in two different chambers thus each volunteer had 20 replicates per treatment per position. **Recaptured–**Total number of mosquitoes caught landing on the body part.

For *An*. *arabiensis* approaching a sleeping volunteer, transfluthrin-treated sandals reduced potential bites over the whole body by 54.1%, without changing the actual distribution of the landing sites (Table 4). Statistically-significant reductions were observed on all exposed body parts except head, torso and upper arms. The transfluthrin-treated sandals had a much greater effect on when the volunteers were standing, as overall reduction of *An*. *arabiensis* landings reached 85.7%. Here, statistically significant reductions occurred everywhere except head, hands and upper arms (Table 4).

**Table 4:** Mean number of *Anopheles arabiensis* caught at different body parts of volunteers with and without the transfluthrin-treated sandals.

		**Volunteers on a sleeping position**							
**Body part**	**N**	**Without sandals**		**With Sandals**		**Paired t-test p-value**		**% Reduction**	
		**Recaptured**	**Mean [95% CI]**	**Recaptured**	**Mean [95% CI]**				
**Head** ^**#**^	40	64	1.6 [1.0–2.2]	105	2.6 [1.8–3.4]	0.0452		-64	
**Hand** *	40	252	6.3 [4.8–7.8]	107	2.7 [1.8–3.6]	<0.001		57	
**Fore arm** ^**#**^	40	471	11.8 [10.1–13.5]	346	8.7 [6.8–10.5]	0.026		27	
**Upper arm**	40	180	4.5 [3.1–5.9]	122	3.1 [2.0–4.1]	0.1353		32	
**Torso**	40	63	1.6 [0.9–2.3]	48	1.2 [0.7–1.7]	0.381		24	
**Upper leg** *	40	317	7.9 [6.1–9.7]	101	2.5 [1.7–3.3]	<0.001		68	
**Lower leg** *	40	467	11.7 [9.6–13.8]	127	3.2 [2.3–4.1]	<0.001		73	
**Foot** *	40	280	7.0 [5.6–8.4]	7	0.2 [0.0–0.3]	<0.001		98	
	N	**Volunteers on standing position**					**Paired t-test p-value**		**% Reduction**
		**Without sandals**		**With Sandals**					
		**Recaptured**	**Mean [95% CI]**	**Recaptured**	**Mean [95% CI]**				
**Head**	40	0	0	2	0.1 [0.0–0.1]	0.1599		_	
**Hand**	40	0	0	2	0.1 [0.0–0.1]	0.1599		_	
**Fore arm**	40	12	0.3 [-0.1–0.7]	9	0.2 [0.0–0.5]	0.7575		25	
**Upper arm**	40	0	0	0	0	_		_	
**Torso**	40	1	0	0	0	_		100	
**Upper leg** ^**++**^	40	73	1.8 [0.6–3.1]	15	0.4 [0.1–0.6]	0.0217		79	
**Lower leg** *	40	718	18.0 [13.3–22.6]	173	4.3 [2.9–5.8]	<0.001		76	
**Foot** *	40	1,605	40.1 [34.4–45.8]	144	3.6 [2.4–4.8]	<0.001		91	

^**#**^ p≤ 0.05

^**++**^ p≤0.01

* p≤0.001. Reporting the two-sided p-value of paired T-test.

**N–**total number of replicates, we had two volunteers in two different chambers thus each volunteer had 40 replicates per position. **Recaptured–**Total number of mosquitoes caught landing on the body part.

## Discussion

Insecticide treated nets and indoor residual sprays can significantly suppress vector populations and the diseases they transmit [2]. However, gaps have been identified in the personal protection they currently confer. For example, these interventions are not effective against exophagic mosquitoes and cannot protect people during waking and active hours [25]. Moreover, their continuous use can cause changes in the feeding patterns and host preferences of mosquitoes [26–30]. These challenges, indicate the need to continue studying the behaviours of vectors and humans, so as to develop complementary measures to curb any persistent transmission. This current study investigated the preferred biting sites by the dengue vector, *Ae*. *aegypti* and the malaria vector, *An*. *arabiensis* on adult volunteers in standing or sleeping positions; and estimated protection limits affordable from protective repellent-treated footwear.

Earlier studies on the biting behaviour of vectors have described the preference of different mosquito species to bite on different parts of human body [11, 31]. For example, *Culex pipiens* mosquitoes were observed to preferentially bite on the lower parts of the body when the host was in sitting position [32] while *Ae*. *aegypti* preferred biting all over the exposed body parts [8]. On the other hand, *Anopheles* mosquitoes, such as those that transmit malaria, typically bite lower parts of the body [11, 12]. Braack *et al* demonstrated that *Anopheles* species appear to bite more on the lower limbs when individuals are standing upright but more evenly distributed over exposed body parts whenever the people are lying down [11]. Sangoro *et al* provides initial evidence that repellent impregnate footwear [22] can provide personal protection against disease transmitting mosquitoes. However this current study found that with transfuthrin treated sandals, the density of *An*. *Arabiensis* in the low limbs reduced by 91% when standing upright and 98% when sleeping. This findings gives important evidence that repellent impregnate footwear [22] can provide personal protection against malaria transmitting mosquitoes (*An*. *Arabiensis)*, even if the sandals do not change the overall distribution of mosquito bites.

Thus, this data is important for advocating the inventions of new interventions that provide personal protections for day biting as well as the night biting mosquitoes. The direct observations of the distribution of bites allows for theoretical estimations of the potential impact of protective clothing, especially those that cover preferred biting areas, such as feet and lower legs. The comparative observations done on both waking and sleeping volunteers also confirms that while the differential proclivity of bites may be useful for designing interventions during waking hours, it is not as useful for sleeping hours.

This study observed that transfluthrin-treated sandals were more effective against malaria vector, *An*. *arabiensis* than against the dengue vector, *Ae*. *aegypti*. They reduced *An*. *arabiensis* bites by 54–86% and *Ae*. *aegypti* bites by 32–39%, but did not change overall distributions of bites. Given that current personal protection against mosquito biting is still challenging especially for outdoors and before bedtime [33], such an intervention could contribute effectively to providing complementary protection at times before people go under their bed nets. Previous study suggested the use of tropical repellent [34] for personal protection, despite the occasional limitations such as requiring multiple applications. The spatial repellent products carrying transfluthrin as the main active ingredient so circumvent this challenge by not requiring regular retreatment. This can be especially useful in locations such as rural Tanzania, where for multiple reasons such as lack of electricity and the types of housing, residents spend significant periods of their time in the evening outdoors doing various activities such as cooking, eating, telling stories and other household activities [35–37]. Such outdoor activities put communities at high risk of getting malaria and other mosquito borne diseases [38], and may be addressed at least in part by interventions such as the repellent-treated sandals.

Perhaps the more direct indication of this study was the potential of personal protection. Based on the distribution of bites, the study theoretical calculations suggest that wearing protective clothing that leave only hands and head uncovered (e.g. socks + trousers + long-sleeved shirts) could prevent 78–83% of all bites during sleeping, and at between 90% and 99.9% of the bites during non-sleeping hours. The evidence also suggests that if the feet are also exposed, the actual levels of protection will decline to as low as 36% against *Anopheles*. It is important therefore that personal protection measures are primarily targeted at the areas where mosquitoes are most likely to bite, especially during waking hours. The advantage of transfluthrin treated sandals is they can offer round the clock protection against the bites. In this case, simple footwear and socks, and where possible the addition of long sleeved clothing and long trousers, can provide significant protection in communities where access to other forms of personal protection is limited. Insecticide-impregnated clothing are already demonstrated to prevent bites, and often require no daily reapplication [39–41]. However, it is likely that even simple physical protection would provide substantial protection already.

It is clear that the repellent sandals were less effective against *Aedes* mosquitoes than against *Anopheles*. However, the distribution of bites suggest that it would still be possible to achieve upwards of 70% protection with just protective clothing. To control the day biting *Aedes*, although topical repellents could be a suitable alternative, these are limited by the fact they need to be reapplied often thus user compliance is attenuated [34, 42, 43]. By combining transfluthrin treated sandals and impregnated clothing will provided a suitable personal protection against *Ae*. *aegypti* which shown to bite at both upper and lower parts of the body [44]. As the malaria and other mosquito borne diseases keep remain a challenge, it is the important to consider these innovations for fighting against mosquito borne diseases.

Stakeholders should consider advocating the scaling up of these innovations to reduce the burden of mosquito borne diseases. Social and behaviour change communication (SBCC) is important tool in advocating malaria control and elimination [45]. Previous studies reported that SBCC has been used to informing malaria surveillance, seasonal variation of malaria cases, treatment management of malaria cases and mosquito borne diseases preventions [46]. Since this study has shown the potential of fighting against mosquito biting by targeting their behaviours, SBCC can also be used to communicate these behaviours and the new mechanisms like the use of transfluthrin treated sandals for personal protection against malaria transmission. However protecting footwear and clothes should consider the climatic conditions of all malaria endemic countries and should not be uniform, because the climatic condition differ from one county to another, the countries with hot climatic condition need to wear open shoes as well as light material clothing compared to cold climate. Also the comfortability of the communities that will be using those interventions should be assessed and communicated earlier before the launching the interventions. However SBCC can be used to assess the need, acceptability and opinions of the communities who are the end users of these interventions. Nevertheless SBCC can be a useful mechanism since it can be used even in hard to reach communities to advocate the new interventions.

## Conclusions

This study adds to the body of evidence on species differences in distribution of landing and biting sites over human bodies when individuals are in the upright position. And shows that when hosts are sleeping, bites from different species might be evenly-distributed over the exposed body surfaces. This study highlights the importance of insecticide treated and untreated personal protection measures in preventing mosquito bites and pathogen transmission from *Anopheline* and *Aedes* mosquitoes. Moreover, that transfluthrin-treated footwear can confer protection against different mosquito species but more against species like *An*. *arabiensis* that prefer to feed on the lower limbs of the human body. Since previous studies also demonstrated *An*. *gambiae* and *An*. *funestus* prefer to bite on the lower parts of the body, especially lower limbs and lower legs, targeted personal protection measures as well as transfluthrin-treated sandals may be potentially effective against these major malaria vectors as well. However, resistance tests should be conducted regularly to ascertain efficacy against the candidate insecticides. Contrarily, simply wearing long-sleeved clothing might suffice in offering the personal protection required. Lastly, using treated personal protection measures on targeted body might shift the biting preferences in some of the host seeking mosquitos as seen for *Ae*. *Aegypti* in this study. Thus one should consider using multiple personal protective measures where possible to ensure the entire body is protected.

## Abbreviations

LLINs: Long- lasting insecticide-treated bed net
IRS: indoor residual spray
ITN: Insecticide treated bed net
PVC: Polyvinyl chloride
SBCC: Social and behaviour change communicatios
